# Epidemiology and management of pediatric femoral fractures at a level I trauma center: a 10-year retrospective monocentric study

**DOI:** 10.1007/s00068-026-03251-z

**Published:** 2026-06-30

**Authors:** Sven-Oliver Dietz, Frank Traub, Elisa Schmidt, Philipp Schippers, Erik Wegner, Lotte Schierjott, Erol Gercek, Tobias Eckard Nowak

**Affiliations:** Departmant of Orthopedics and Traumatology, University Medical Center of the Johannes Gutenberg University Mainz, Mainz, Germany

**Keywords:** Pediatric trauma, Femur fracture, Epidemiology, ESIN, Complications

## Abstract

**Purpose:**

Pediatric femoral fractures are relatively uncommon but represent severe injuries frequently requiring inpatient trauma care. Epidemiological characteristics, fracture patterns, and treatment strategies vary between institutions and regions. This study aimed to analyze the epidemiology, fracture distribution, treatment modalities, complications, and fracture-predisposing comorbidities of pediatric femoral fractures treated at a German level I pediatric trauma center over a 10-year period.

**Methods:**

All patients aged 0–14 years treated for femoral fractures between January 2012 and December 2021 were retrospectively reviewed. Fractures were categorized using the AO Pediatric Comprehensive Classification of Long-Bone Fractures (PCCF). Demographic variables, fracture characteristics, management approaches, time to consolidation, complications, and relevant comorbidities predisposing to fracture were analysed descriptively in accordance with STROBE recommendations.

**Results:**

A total of 183 patients met the inclusion criteria (67.2% male; mean age 5.2 years). Children aged 2–5 years constituted the largest group (42.1%). Femoral shaft fractures were the most frequent femoral fracture type (70%). Surgical management, most frequently elastic stable intramedullary nailing (ESIN), was increasingly utilized with advancing age. The overall complication rate was 14.2%, with the majority classified as minor. Comorbid conditions predisposing to fracture were present in 22% of patients.

**Conclusion:**

Femoral shaft fractures represented the predominant femoral fracture type in this trauma-center cohort, particularly affecting boys in early childhood. Surgical stabilization represents an integral component of contemporary pediatric trauma care and was associated with acceptable, predominantly minor complication rates. This study contributes contemporary real-world epidemiological data from a level I pediatric trauma reference center and highlights relevant age-dependent treatment trends and clinical outcomes.

## Introduction

Pediatric femoral fractures account for a small proportion (1–3%) of pediatric fractures but pose a substantial challenge in trauma and emergency treatment due to prolonged hospitalization, immobilization, and potential long-term sequelae [1–3]. With an incidence of approximately 11.2 per 100,000 children, these injuries are clinically relevant as they are frequently associated with high-energy mechanisms, polytrauma, pathological fractures, or suspected non-accidental injury, particularly in infants and toddlers [4–6]. Previous studies have consistently demonstrated a predominance of male patients, with femoral shaft fractures representing the most frequent fracture pattern [2, 7, 8]. However, reported age distributions, fracture subtypes, and treatment strategies vary considerably between population-based registries and monocentric trauma center cohorts [4, 7]. Conservative treatment, using spica-cast and/or overhead-extension, has been and continues to be the preferred option for children under the age of 3 due to the high potential for remodelling and rapid consolidation, despite potential growth disturbances caused by the residual axial deviations [9]. Nevertheless, over recent decades, treatment paradigms have shifted toward early surgical stabilization, most commonly using elastic stable intramedullary nailing (ESIN), even in children younger than 3 years of age [10–12]. The German guideline already points out that, in “individual cases, [surgical treatment] may also be used in children under the age of 3” [13]. A survey conducted by the German Society for Trauma Surgery revealed that half of all participating clinicians in Germany also perform surgical treatment on children under the age of 3 [14]. Despite a substantial body of literature on pediatric femoral fractures, important gaps remain regarding real-world treatment patterns and outcomes in tertiary trauma care settings. In particular, most available data are derived from population-based registries or heterogeneous multicenter cohorts, which may not adequately reflect the complexity of patients treated in specialized trauma centers, including children with fracture-predisposing comorbidities.

The present study provides a comprehensive single-center analysis from a level I pediatric trauma center, offering detailed insights into age- and sex-specific fracture distribution, contemporary treatment strategies across different anatomical regions, and complication profiles in a real-world clinical environment. By explicitly including patients with underlying conditions affecting bone quality, this study further addresses an underrepresented but clinically highly relevant subgroup.

These findings contribute to a more nuanced understanding of current treatment practices and their outcomes, and may support more individualized, risk-adapted decision-making in pediatric femoral fracture management.

The aim of this study was to provide a comprehensive epidemiological assessment of pediatric femoral fractures treated at a German level I pediatric trauma center over a 10-year period, with particular emphasis on fracture characteristics, treatment strategies, and complications, and to contextualize the findings within the existing trauma literature.

## Materials and methods

### Study design and setting

A retrospective, single-center cohort study was conducted at a level I pediatric trauma center. All pediatric patients treated for femoral fractures between January 1, 2012 and December 31, 2021 were eligible for inclusion. The institutional age limit for pediatric trauma care was 14 years.

### Study population

All patients aged 0–14 years with radiographically confirmed femoral fractures who were primarily treated at our institution during the study period were included.

Exclusion criteria comprised incomplete medical records, patients who received primarily treatment at another institution with subsequent secondary referral only, and pathological fractures related to malignant disease.

### Data sources and data collection

Data were retrospectively extracted from electronic medical records, operative reports, outpatient follow-up documentation, and radiological archives using a standardized data extraction form. Demographic data included age at injury and sex. Fracture characteristics comprised fracture location (proximal femur, femoral shaft, distal femur) and fracture morphology.

Fractures were classified according to the AO Pediatric Comprehensive Classification of Long-Bone Fractures (PCCF). Treatment modality (conservative or surgical), specific surgical techniques, time to radiographic consolidation, and implant removal were documented.

Fracture-predisposing comorbidities were defined as underlying conditions associated with impaired bone quality or increased fracture risk, including syndromic disorders, metabolic bone disease, benign bone lesions, and neuromuscular disorders.

### Treatment strategies

Treatment strategies were categorized as conservative or surgical. Due to the retrospective design, no predefined treatment algorithm was applied.

Conservative management included spica casting, overhead extension, and functional immobilization, depending on patient age, comorbidities, fracture pattern, and clinical stability.

Surgical treatment comprised elastic stable intramedullary nailing (ESIN), plate osteosynthesis, external fixation, screw fixation, K-Wire fixation and additional stabilization techniques such as cerclage wiring where indicated. The choice of treatment was based on fracture configuration, patient age, body weight, and the presence of comorbidities, in accordance with institutional standards and current guidelines.

Implant removal was performed after confirmed bone consolidation in accordance with institutional standards, typically after approximately 6 months for ESIN and 12 months for plate fixation.

### Outcomes and definitions

Bone consolidation was defined as a callus spanning ≥ three cortices.

Complications were defined as any adverse event related to the fracture or its management occurring during treatment or follow-up. Complication severity was stratified using the Clavien–Dindo classification, with complications not requiring invasive intervention classified as minor (Clavien–Dindo ≤ II) and events necessitating surgical or procedural intervention classified as major (Clavien–Dindo ≥ III) [15].

Rotational malalignment was assessed clinically and radiographically based on documented follow-up examinations. Fracture-predisposing comorbidities were documented separately.

Complications were recorded during the in-hospital stay and available follow-up period.

### Bias and study limitations

Due to the retrospective and monocentric design, selection bias and information bias cannot be excluded. The study cohort reflects a tertiary care trauma population, which may limit generalizability to other healthcare settings. To minimize classification bias, fractures were categorized using a standardized and validated pediatric classification system (AO PCCF).

## Study size

All eligible patients treated during the 10-year study period were included. No formal sample size calculation was performed owing to the descriptive and exploratory nature of the study.

### Statistical analysis

Data were analyzed descriptively. Categorical variables are presented as absolute numbers and percentages, and continuous variables as means or medians with ranges as appropriate.

No inferential statistical analyses were performed, as the primary objective of the study was to provide a comprehensive epidemiological overview rather predefined hypotheses.

### Missing data

Missing data were rare and did not exceed 5% for any key variable. Cases with incomplete documentation of key variables were excluded from the final analysis. No imputation was performed.

### Ethical considerations and consent to participate

The study was approved by the local institutional ethics committee (approval number: 2025–18137). Due to the retrospective design of the study and the use of fully anonymized data, the requirement for informed consent to participate was waived by the responsible institutional ethics committee in accordance with national regulations.

### Reporting guidelines

The study was conducted and reported in accordance with the Strengthening the Reporting of Observational Studies in Epidemiology (STROBE) guidelines for observational studies [16].

## Results

We searched our digital patient database for the period from January 2012 to December 2021 for all patients (without age restriction) with ICD codes S72.0x–S72.9. A total of *n* = 2981 patients matched these codes, of whom *n* = 203 were eligible for inclusion. Patients with incomplete data (*n* = 20) were excluded. 183 Patients were included in the final analysis.

### Sex distribution and fracture location frequency

Of the 183 included patients, 123 were male (67.2%) and 60 female (32.7%), yielding a male-to-female ratio of 2.1:1. Overall, femoral shaft fractures constituted the most frequent femoral fracture type (70%), followed by distal femoral fractures (20%) and proximal femoral fractures (10%).

### Sex-specific differences in fracture localization

**Fig. 1:**
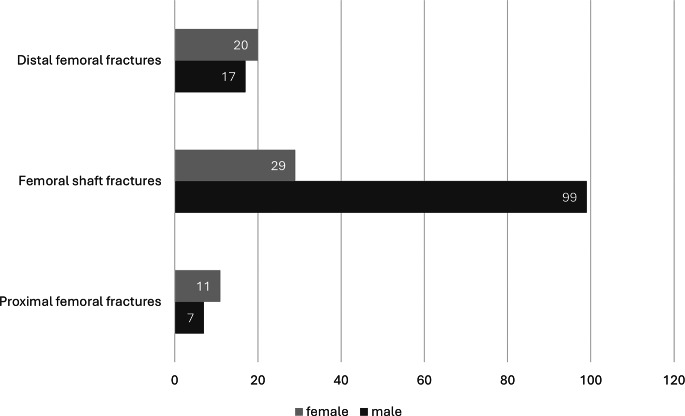
Sex distribution according to femoral fracture location

Sex-specific analysis revealed distinct differences in fracture localization. Among male patients, femoral shaft fractures predominated (*n* = 99; 80.4%), whereas distal (*n* = 17; 13.8%) and proximal femoral fractures (*n* = 7; 5.6%) were less common. In contrast, female patients demonstrated a more heterogeneous distribution, with *n* = 29 (48.4%) sustaining femoral shaft fractures, *n* = 20 (33.3%) distal femoral fractures, and *n* = 11 (18.3%) proximal femoral fractures (Fig. 1).

### Age distribution

**Fig. 2:**
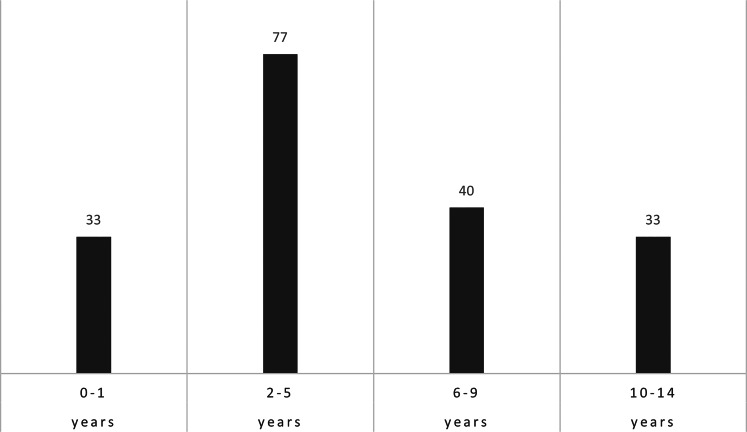
Overall age distribution of the study population

The mean age of the cohort was 5.2 years (range: 4 weeks to 14 years). The highest incidence of femoral fractures was observed in children between 2 and 5 years (*n* = 77; 42.1%), followed children aged 6–9 years (*n* = 40; 21.8%). Children 0–1 years and 10–14 years each accounted for 18% (*n* = 33) of cases (Fig. 2).

### Proximal femoral fractures

Proximal femoral fractures were rare, occurring in 18 patients (10%). The highest incidence was observed in adolescents aged 10–14 years (*n* = 8; 44.4%), with an equal sex distribution. In children aged 6–9 years, proximal femoral fractures were identified in 5 cases (27.8%) and occurred exclusively in girls. In younger age groups proximal fractures were uncommon.

**Fig. 3:**
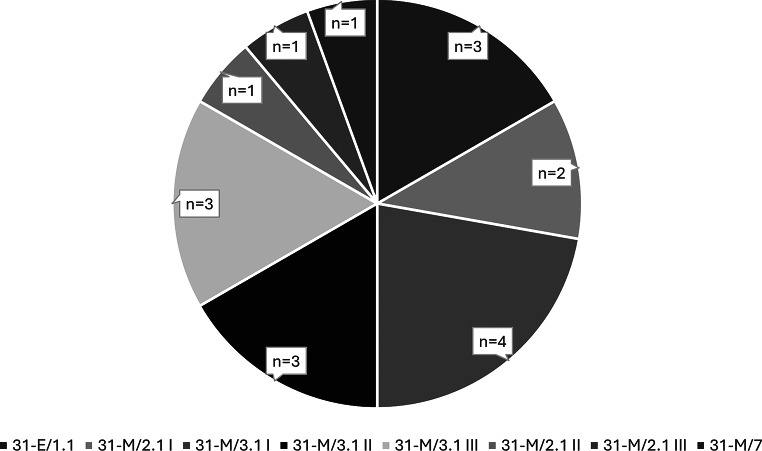
Distribution of proximal femoral fracture types according to the AO PCCF

Fracture patterns were heterogeneous. Femoral neck fractures (31-M/3.1 I) constituted the most frequent subtype (22%; *n* = 4), followed by basocervical femoral neck fractures (31-M/3.1 II), transtrochanteric fractures (31-M/3.1 III), and epiphyseal fractures (31-E/1.1), each accounting for 17% (*n* = 3). All remaining fracture subtypes observed only sporadically (Fig. 3).

Surgical treatment was performed in 13 patients, while 5 children were managed conservatively.

Among surgically treated patients, cannulated screw fixation was the most frequently employed technique (*n* = 7). Cannulated screws with a diameter of 4.5 mm were used in *n* = 5 patients, and those with a diameter of 6.5 mm were used in *n* = 2 patients. Additional surgical procedures included the use of single cannulated screws (*n* = 2), which are commonly used in slipped capital femoral epiphysis (SCFE) fixation. In the present study, these implants were exclusively applied for traumatic proximal femoral fractures (AO 31-E/1.1 and 31-E/2.1). No cases of slipped capital femoral epiphysis were included in this cohort. Pediatric hip plate fixation (*n* = 2), Targon FN^®^ fixation (*n* = 1**)** were used as plate osteosynthesis. The diameter of the Kirschner wires (*n* = 1) was 3.0 mm.

**Fig. 4:**
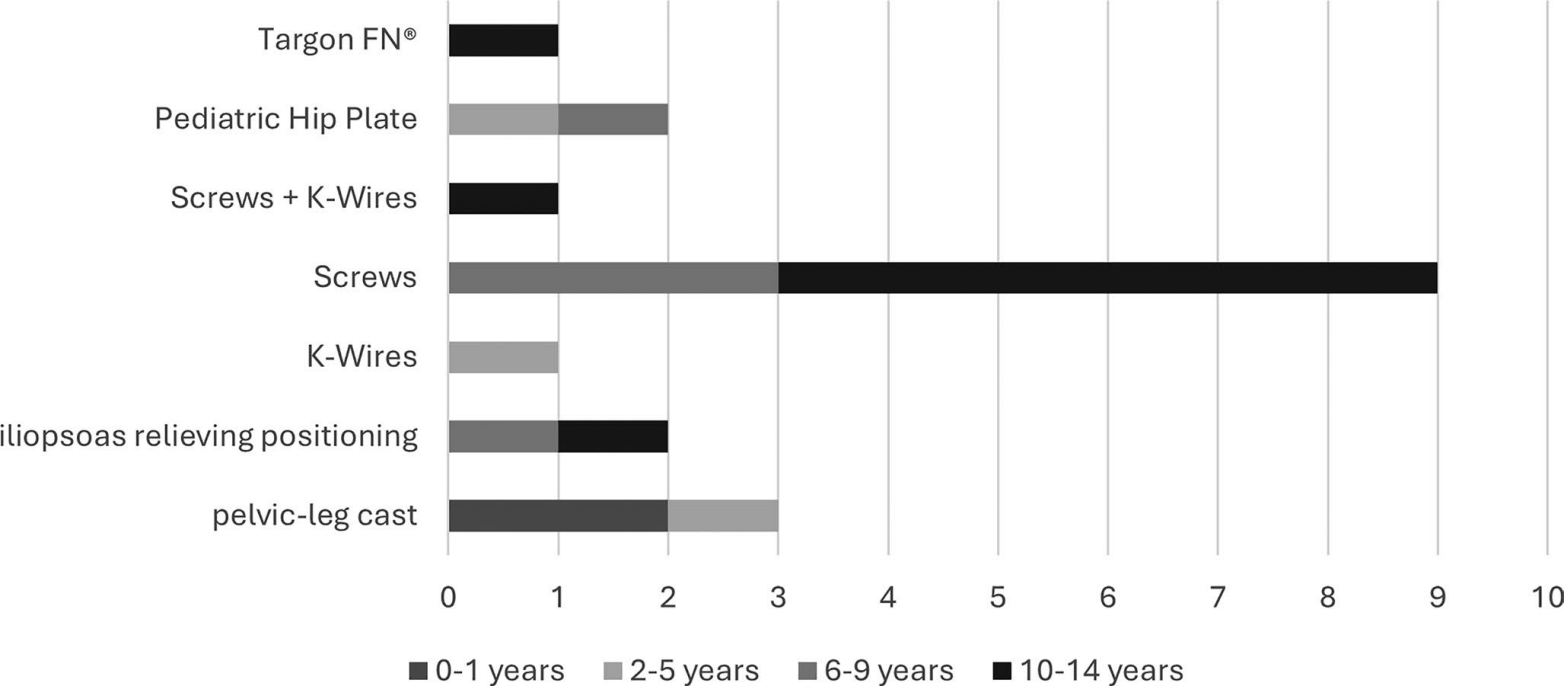
Treatment modality by age group in proximal femoral fractures

Conservative treatment was predominantly applied in younger patients. Three children were treated with a hip-spica cast, including two infants aged 0–1 years and one child aged 5 years. In two older children (aged 6–9 and 10–14 years), for whom surgical intervention was deemed high risk due to underlying conditions, immobilization was not required, and management consisted of analgesia and positioning measures only. Overhead extension was not utilized in any patient within this cohort (Fig. 4).

### Femoral shaft fractures

Femoral shaft fractures occurred most frequently in children aged 2–5 years (*n* = 59; 46.0%), with a pronounced male predominance (48 males vs. 11 females). The second most affected group comprised children aged 6–9 years (*n* = 28; 21.9%). In infants aged 0–1 years, 21 femoral shaft fractures (17.5%) were documented, while 20 cases (15.6%) occurred in children aged 10–14 years. Female patients were underrepresented at the extremes of age, with only 3 girls affected in infancy and 6 in the oldest age group.

**Fig. 5:**
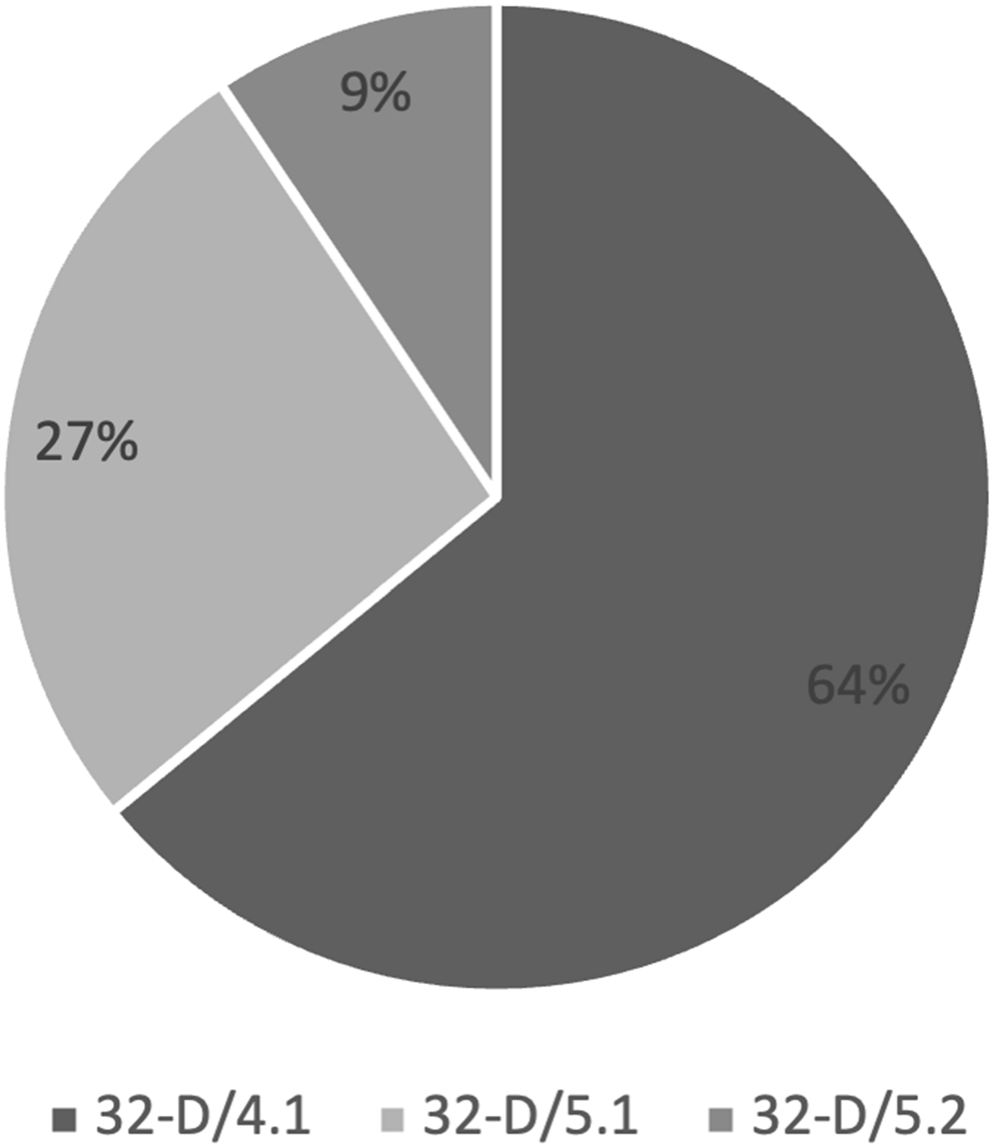
Distribution of femoral shaft fracture types according to the AO PCCF

According to the AO Pediatric Comprehensive Classification of Long-Bone Fractures (PCCF), simple transverse diaphyseal fractures (32-D/4.1) constituted the most common subtype, accounting for 64% (*n* = 82) of femoral shaft fractures. Simple oblique or spiral fractures (32-D/5.1) represented 27% (*n* = 34), whereas multifragmentary oblique or spiral fractures (32-D/5.2) were rare (9%; *n* = 12) (Fig. 5).

Femoral shaft fractures were predominantly treated surgically, with 102 of 128 patients undergoing operative stabilization, whereas 26 children received conservative treatment.

Elastic stable intramedullary nailing (ESIN) was the most frequently applied surgical technique and was used in 89 patients. The majority of these children were 2–5 years of age, including 54 patients, with a peak incidence at 2 years (*n* = 15) and 3 years (*n* = 22). Notably, two children treated with ESIN were younger than 1 year at the time of surgery. In the age group 10–14 years, 11 patients underwent ESIN stabilization. In the 0–1-year-old age group, ESINs of thicknesses 2.0 and 2.5 were each used once. For children aged 2–5 and 6–9 years, all ESINs had a diameter of 2.5 mm. For children aged 10–14 years, the average ESIN diameter was 3.15 mm, with a minimum of 2 mm and a maximum of 4 mm in one patient each. ESINs were inserted antegradely in 2 patients and retrogradely in 87 patients. End caps were used in 37 patients with retrograde ESINs. All patients in whom end caps were used had a AO 32-D/5.1 fracture.

In selected cases, ESIN was combined with external fixation (*n* = 3).

External fixation as a standalone treatment was used in three patients aged 5, 6, and 7 years, respectively. Plate osteosynthesis was performed in 14 patients, predominantly in children aged 10–14 years (*n* = 8). The remaining patients treated with plate fixation included three girls aged 8 years, one boy aged 7 years, and two children aged 5 years. Straight LCPs^®^ (Synthes, Umkirch, Germany) were used in 8 children, whilst long Pediatric Hip Plates^®^ (Synthes, Umkirch, Germany) were used in 6 children with proximal femoral shaft fractures. Frontal axis alignment was assessed in all patients treated with plate osteosynthesis. Following consolidation of the fractures treated with plates, the mechanical femoral axis averaged 6.1°, and the anatomical tibio-femoral angle averaged 175.9°. Both values fall within the normal range and no clinically relevant frontal axis deviations were observed.

**Fig. 6:**
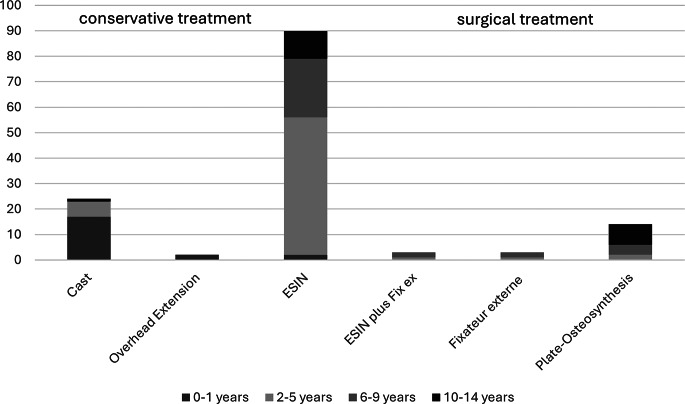
Treatment modalities by age group in femoral shaft fractures

Among patients managed conservatively, 24 children were treated with hip-spica casting, the majority of whom were infants aged 0–1 years (*n* = 17). One child received traction treatment (Fig. 6).

### Distal femoral fractures

Distal femoral fractures (*n* = 37) occurred most frequently in children aged 2–5 years (*n* = 15; 40.6%), with a slight male predominance. In contrast, among infants aged 0–1 year, distal femoral fractures were more frequently observed in girls.

**Fig. 7:**
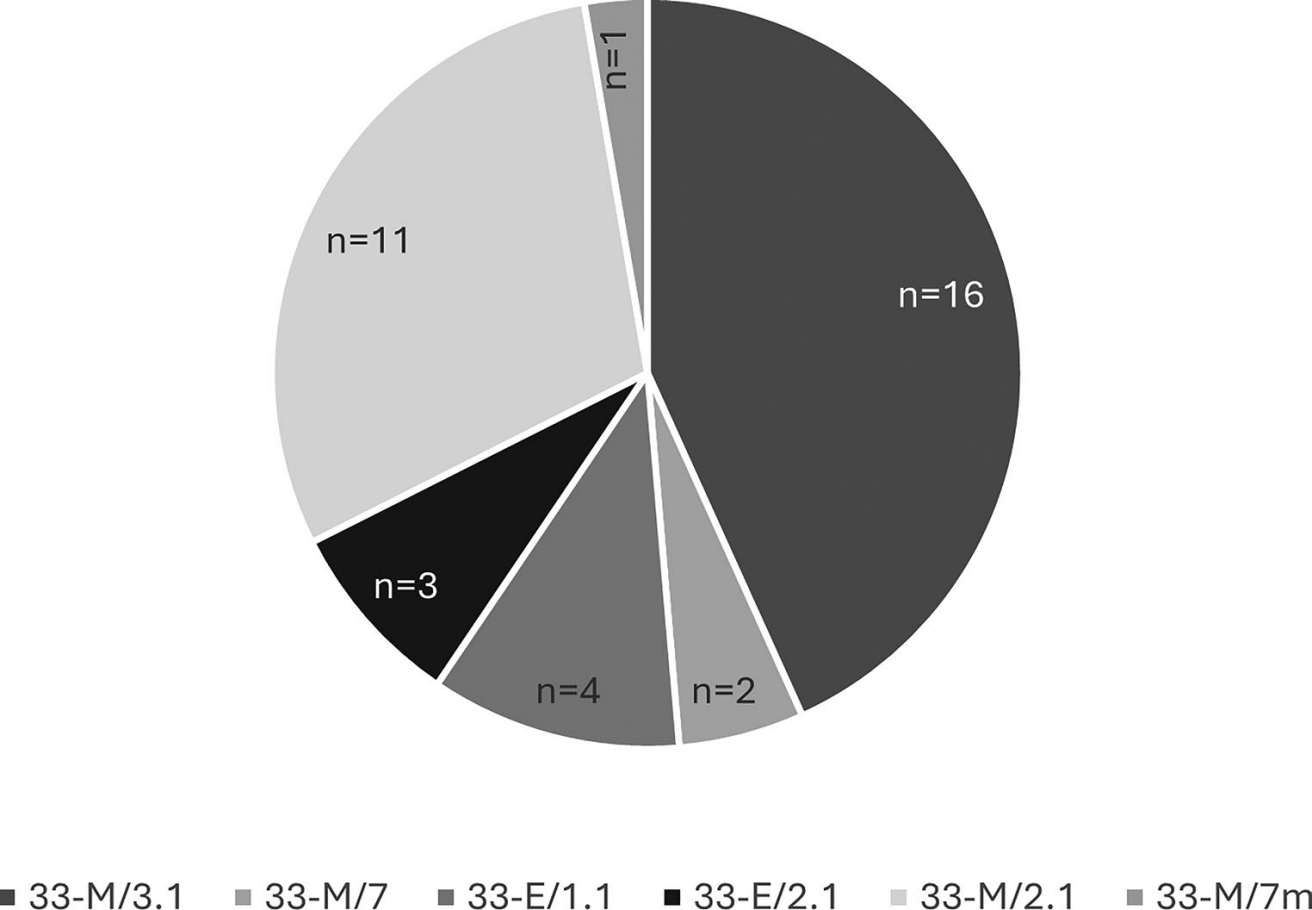
Distribution of distal femoral fracture types according to the AO PCCF

With respect to fracture morphology, complete transverse metaphyseal fractures (33-M/3.1) were the most common distal fracture type (43%; *n* = 16). Torus fractures (33-M/2.1) accounted for 30% (*n* = 11), while Salter–Harris type I epiphyseal fractures (33-E/1.1) were identified in 11% (*n* = 4) of cases. Other distal fracture subtypes were rare and occurred only sporadically (Fig. 7). The observed distribution should be interpreted in the context of the institutional age limit of 14 years.

**Fig. 8:**
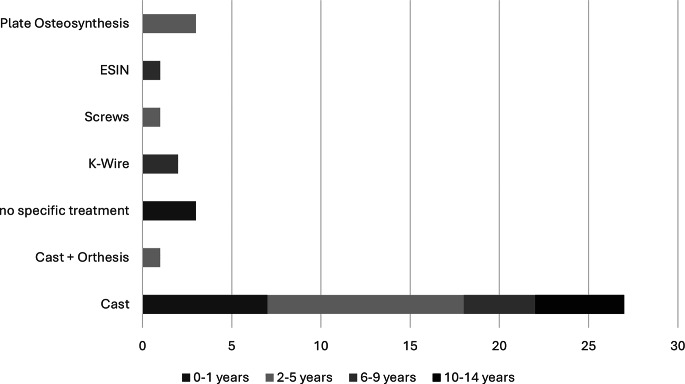
Treatment modalities by age group in distal femoral fractures

Distal femoral fractures were most frequently managed conservatively (*n* = 30; 81.1%), while 7 patients (18.9%) underwent surgical stabilization. Surgical techniques included plate osteosynthesis (*n* = 3), K-wire osteosynthesis (*n* = 2), elastic stable intramedullary nailing (ESIN) (*n* = 1), and cannulated screw fixation (*n* = 1). Among conservatively treated patients, 26 children received immobilization using a cast or splint, most commonly in children aged 2–5 years (*n* = 10) and infants aged 0–1 years (*n* = 7). Additional conservative measures included abduction orthosis application in one two-year-old child, analgesic treatment without immobilization in three patients, and compression bandaging alone in one patient (Fig. 8).

### Comorbidities associated with femoral fractures

Comorbidities potentially predisposing to femoral fractures were identified in 41 patients (22%). The most common conditions were severe syndromic disorders (*n* = 17) and bone cysts (*n* = 6), followed by non-ossifying fibromas (*n* = 5). Musculoskeletal disorders associated with impaired bone quality were rare and occurred only sporadically, including osteogenesis imperfecta (*n* = 2), osteoporosis–pseudoglioma syndrome, spinal muscular atrophy, and fibrous dysplasia (*n* = 1 each). No association between fracture location and comorbidities could be established.

### Treatment strategies and fracture consolidation

Treatment strategies varied according to patient age and fracture localization. Across all fracture types, conservative treatment predominated in infants, whereas surgical stabilization was increasingly applied with advancing age. The distribution of conservative and operative treatment strategies for proximal, shaft, and distal femoral fractures is illustrated in Figs. 4, 6 and 8.

For femoral shaft fractures, surgical stabilization was most performed using elastic stable intramedullary nailing (ESIN). The median time to radiographic fracture consolidation was 8 weeks following surgical treatment and 4 weeks after conservative management. Implant removal was performed after a median of 6 months. The timing of implant removal varied across fracture locations, reflecting differences in healing characteristics and individual patient factors. In proximal femoral fractures, implant removal was generally performed later, most commonly between 7 and 12 months, with some cases extending beyond 15 months. In femoral shaft fractures, implant removal was predominantly performed within the first 6 months, although a substantial proportion of patients underwent removal between 7 and 12 months. In distal femoral fractures, implant removal was performed between 4 and 24 months, with variability influenced by individual clinical circumstances. In a subset of patients with radiologically confirmed bone union, implant removal was not performed at our institution.

### Complications

Overall, complications occurred in 26 of 183 patients (14.2%). Most complications were classified as minor and did not require surgical revision. No neurovascular complications or deep infections were observed. Minor superficial skin irritation related to implants was recorded.

After surgical treatment, 15 minor complications were observed. Among these, 11 patients experienced soft tissue irritation caused by the osteosynthesis material. Four cases of minor rotational malalignment (< 10°) were documented. All rotational deviations were managed conservatively and resolved spontaneously during follow-up without the need for revision surgery.

In addition, three major complications following ESIN osteosyntheses requiring revision surgery were identified. All of these occurred in patients with severe underlying disorders affecting bone metabolism and healing capacity. One revision involved a 6-year-old boy with osteoporosis–pseudoglioma syndrome (OPPG), who exhibited severely impaired bone healing due to disturbed bone metabolism and pronounced immobility. Owing to implant failure, secondary displacement, and persistent pain, a total of four revision procedures with interim cast immobilization were required to achieve acceptable fracture consolidation. A second case concerned a 13-year-old boy with cranio-carpo-tarsal syndrome (CASH), who developed delayed union and instability after ESIN fixation. Adequate consolidation was ultimately achieved after the third revision surgery, including conversion to angular-stable plate fixation, cancellous bone grafting, and additional cast immobilization. Due to the underlying disease and persistently poor bone quality, implant removal was intentionally omitted. The third revision occurred in an 11-year-old child with osteogenesis imperfecta and a history of prior femoral fracture treated abroad. Following a recurrent femoral shaft fracture managed with titanium elastic nailing, revision surgery became necessary because of insufficient implant anchorage and compromised bone quality.

Following conservative treatment, four minor soft-tissue complications and three cast-related complications requiring cast replacement were documented. One re-fracture occurred after conservative treatment, which was associated with a new traumatic event following discharge and was successfully treated conservatively without the need for surgical intervention.

## Discussion

This study provides contemporary trauma-center-based epidemiological data on pediatric femoral fractures.

### Age and sex distribution

Consistent with previous epidemiological studies, femoral fractures predominantly affected boys, with a male-to-female ratio of approximately 2:1 [1, 3, 7, 8, 17]. The mean age of 5.2 years and the peak incidence observed in preschool-aged children (2–5 years) are in close agreement with findings from prior studies [7, 10].

In contrast to the frequently reported bimodal age distribution with an additional peak during adolescence [12, 18–20], no second peak was observed in the present study. This finding is most likely attributable to the upper age limit of 14 years and the monocentric trauma-center setting, as similarly reported in comparable cohorts [12, 19, 20].

### Fracture localization and patterns

Femoral shaft fractures represented the most common fracture type across all age groups, confirming previous reports that diaphyseal fractures account for most pediatric femoral injuries [2, 8, 20, 21]. The predominance of simple transverse diaphyseal fractures observed in our cohort is consistent with findings by Flynn et al. [18], but contrasts with studies identifying spiral fracture patterns as most frequent [22, 23]. These discrepancies likely reflect population-specific differences in injury mechanisms and trauma exposure.

Distal femoral fractures constituted the second most common localization in the present cohort, accounting for 20% of all femoral fractures, which is within the range reported in the literature [8, 20, 24]. While Engström et al. identified Salter–Harris type II injuries as the most frequent distal femoral fracture subtype [21], metaphyseal fractures predominated in our study. This divergence may be attributable to the high proportion of children with severe syndromic disorders and impaired bone metabolism in our cohort, predisposing them to low-energy metaphyseal fractures. The distal femoral physis remains open until late adolescence, which has important implications for fracture patterns and treatment strategies. The institutional age limit of 14 years in the present study may therefore have led to an underrepresentation of adolescent physeal injuries, potentially influencing the observed epidemiological distribution.

Proximal femoral fractures were rare (10% of cases), in line with previous reports [7, 20, 21, 25]. As expected, proximal fractures occurred more frequently in older children and demonstrated a heterogeneous fracture pattern. The distribution of femoral neck fracture subtypes was comparable to previous studies and aligned with the Delbet–Basset–Collona classification, thereby supporting the comparability of our findings with existing literature [25, 26].

### Treatment strategies

Treatment strategies in the present cohort were consistent with current clinical guidelines, with femoral shaft fractures predominantly managed surgically using ESIN, particularly in children older than three years [10, 27, 28]. The high proportion of ESIN-treated patients reflects the global trend toward operative stabilization, driven by shorter hospitalization, earlier mobilization, and reduced caregiver burden [14, 27].

Although conservative treatment remains the standard treatment for infants younger than 3–5 years due to the high remodeling potential of the growing skeleton [14, 29], the present study demonstrates an increasing use of ESIN even in children younger than 3 years. This observation is consistent with recent reports describing the safe application of ESIN in carefully selected children younger than 3 years. The use of ESIN in children under 1 year of age remains controversial, as conservative treatment is generally recommended due to the high remodeling potential. In the present study, surgical stabilization was performed in an individual case based on fracture characteristics and clinical considerations. While parental preference contributed to the decision-making process, the indication for surgery was established after careful evaluation of the risks and benefits. This reflects the need for individualized treatment decisions in selected cases, as also discussed in recent literature [14, 28, 30]. Treatment decisions in our cohort were individualized, taking fracture stability, patient age, body weight, and comorbidities into account, in accordance with current recommendations [13, 31].

Distal femoral fractures were predominantly managed conservatively, particularly in stable and non-displaced fracture patterns, consistent with previous reports [21, 24]. Operative stabilization was reserved for displaced fractures, cases with compromised perfusion, or older children at increased risk of malalignment. Particularly Salter–Harris type II injuries of the distal femur are associated with a considerable risk of growth disturbances, as described in previous studies [32–34]. Proximal femoral fractures were more frequently treated with surgical stabilization favored especially in displaced fractures and older children owing to the increased risk of secondary displacement and avascular necrosis [25, 26].

While the increasing use of surgical stabilization offers several advantages, including earlier mobilization and reduced hospitalization, it must be balanced against procedure-related risks. In particular, surgical treatment requires general anesthesia, which is associated with inherent risks, especially in very young children. Although serious anesthesia-related complications are rare, this aspect should be considered when weighing operative versus conservative management. Therefore, careful patient selection and individualized risk–benefit assessment remain essential.

### Complications

Complications occurred primarily after femoral shaft fractures and were predominantly minor. Major complications requiring revision surgery were observed almost exclusively in patients with impaired bone quality, consistent with previous reports [35, 36]. No deep infections or neurovascular injuries were identified in the present cohort.

Although the overall complication rates after operative and conservative treatment were comparable, the nature of complications differed. Iatrogenic complications associated with ESIN were predominantly technical and potentially avoidable, underscoring the importance of surgical expertise, appropriate nail diameter selection, and correct implant positioning [12, 37–39].

In contrast to reports suggesting higher complication rates following operative treatment in children younger than three years [12], age was not identified as an independent risk factor for complications in the present cohort. Instead, complications were more frequently with underlying comorbidities and increased fracture complexity. These findings suggest that, in experienced hands, ESIN can be a safe treatment option even in selected very young children, as also reported by Strohm et al. and others [14, 30].

The assessment of rotational malalignment in pediatric patients is subject to inherent limitations, particularly for small angular deviations. Clinical and radiographic measurements may be affected by interobserver variability, and minor rotational differences are difficult to quantify reliably. In the present study, all observed rotational deviations were minor (< 10°), clinically asymptomatic, and resolved without intervention, suggesting limited clinical relevance.

### Clinical implications

The high rate of surgical stabilization in the present cohort reflects the ongoing shift toward early operative management in pediatric trauma care. The predominance of minor complications observed is consistent with previously reported outcomes following elastic stable intramedullary nailing in appropriately selected pediatric patients [10–12, 40]. Nevertheless, careful patient selection remains essential, particularly in children with severe comorbidities and compromised bone quality. In addition, the risks associated with general anesthesia should be considered, particularly in very young children, reinforcing the importance of individualized treatment decisions.

## Limitations

This study has several limitations that should be considered when interpreting the results.

First, the retrospective study design inherently carries a risk of incomplete or inconsistent documentation and limits control over potential confounding variables. Although data were extracted from comprehensive electronic medical records, minor complications or subtle functional deficits may have been underreported.

Second, the monocentric design and conduct of this study and its conduct at a level I pediatric trauma center introduce a potential selection bias toward more complex injuries and patients with severe comorbidities. Consequently, the observed fracture patterns, treatment strategies, and complication rates may not be fully generalizable to population-based cohorts or lower-level trauma centers.

Third, the analysis was intentionally descriptive. Given the heterogeneous distribution of fracture types, age groups, and treatment modalities, no inferential statistical analyses were performed. As a result, causal inferences regarding risk factors for complications or superiority of specific treatment strategies cannot be drawn.

Fourth, a relevant proportion of major complications occurred in patients with severe fracture-predisposing comorbidities associated with impaired bone metabolism. Although these cases reflect real-world clinical practice in a tertiary referral center, they may have disproportionately influenced overall complication and revision rates.

Fifth, standardized functional outcome measures and long-term follow-up data were not consistently available and could therefore not be analyzed. Consequently, functional recovery, limb length discrepancy, and late sequelae such as growth disturbances may be underestimated.

Sixth, the upper age limit of 14 years may have contributed to the absence of a second incidence peak during adolescence, as older adolescents with femoral fractures are frequently treated in adult trauma units.

Another limitation of this study is the lack of systematic data on body weight or body mass index (BMI) at the time of treatment. These factors may influence both treatment selection and complication risk, particularly with regard to implant choice and mechanical stability. Future studies should incorporate weight-related parameters to allow for a more detailed risk stratification.

Finally, the Clavien–Dindo classification was originally developed for general surgical complications and has not been specifically validated for pediatric orthopedic trauma. However, it was used to allow standardized severity stratification.

## Conclusion

Femoral shaft fractures were the most frequent femoral fracture type in children treated at a trauma reference center, predominantly affecting boys aged 2–5 years. Surgical stabilization, particularly elastic stable intramedullary nailing, is an integral component of contemporary pediatric trauma care and was associated with acceptable complication rates, with the majority being minor events.

However, conservative treatment remains an established and effective option in selected pediatric patients, particularly in younger children, and was not specifically evaluated as a comparative strategy in the present study. The relatively high rate of surgical management in this cohort likely reflects the characteristics of a level I trauma center and should be interpreted in this context.

By providing detailed real-world epidemiological data from a level I trauma center, this study enhances the understanding of current treatment patterns and complication profiles in pediatric femoral fractures and supports the implementation of individualized, age-adapted treatment strategies.

## Data Availability

The datasets generated and/or analyzed during the current study are available from the corresponding author on reasonable request.

## References

[1] Nakaniida A, Sakuraba K, Hurwitz EL. Pediatric orthopaedic injuries requiring hospitalization: epidemiology and economics. J Orthop Trauma [Internet]. 2014;28:167–172 [cited 2026 Jan 10]. 10.1097/BOT.0B013E318299CD20. 23681411 10.1097/BOT.0b013e318299cd20

[2] Hedlund R, Lindgren U. The incidence of femoral shaft fractures in children and adolescents. J Pediatr Orthop [Internet]. 1986;6:47–50. [cited 2026 Jan 10]. 10.1097/01241398-198601000-00010. 3941180 10.1097/01241398-198601000-00010

[3] von Heideken J, Svensson T, Blomqvist P, Haglund-Åkerlind Y, Janarv P-M. Incidence and trends in femur shaft fractures in Swedish children between 1987 and 2005. J Pediatr Orthop. 2011;31:512–519. 10.1097/BPO.0B013E31821F9027. 10.1097/BPO.0b013e31821f9027 21654458

[4] Fernandez FF, Eberhardt O. Klassifikationen von frakturen im kindesalter. Trauma Berufskrankh. 2010;12:323–8. 10.1007/s10039-009-1586-y.

[5] Lee Y, Lim K, Gao GX, Mahadev A, Lam KS, Tan SB et al. Traction and spica casting for closed femoral shaft fractures in children. J Orthop Surg. 2007. 10.1177/230949900701500109 17429115

[6] Maguire S, Cowley L, Mann M, Kemp A. What does the recent literature add to the identification and investigation of fractures in child abuse: an overview of review updates 2005–2013. Evid Based Child Health. 2013;8:2044–57. 10.1002/ebch.1941.

[7] Loder RT, O’Donnell PW, Feinberg JR. Epidemiology and mechanisms of femur fractures in children. J Pediatr Orthop [Internet]. 2006;26:561–566 [cited 2026 Jan 10]. 10.1097/01.BPO.0000230335.19029.AB. 16932091 10.1097/01.bpo.0000230335.19029.ab

[8] Kraus RSD. Häufigkeit von Frakturen der langen Röhrenknochen im Wachstumsalter. Dtsch Arztebl. 2005;102:838–42.

[9] Kertai M. Behandlung der Femurschaftfraktur im Wachstumsalter: Vom Beckenbeingips bis zur elastisch stabilen intramedullären Nagelung. Trauma Berufskrankh. 2018. Springer. 10.1007/s10039-017-0269-3.

[10] Kertai M. Behandlung der Femurschaftfraktur im Wachstumsalter. Trauma Berufskrankh [Internet]. 2018;20:1–5. 10.1007/s10039-017-0269-3.

[11] Rether JR. Intramedulläre Stabilisierung von Schaftfrakturen im Wachstumsalter. Trauma Berufskrankh. 2005;7:112–7. 10.1007/s10039-005-1004-z.

[12] Oberthür S, Piatek S, Krause H, Rüther H, Roch PJ, Zoch A, et al. Complication rate after femoral shaft fractures in childhood and adolescence depending on patient factors and treatment measures. Chirurg Springer Medizin. 2022;93:165–72. 10.1007/s00104-021-01437-2. 10.1007/s00104-021-01437-2 PMC8821081 34132823

[13] Deutsche Gesellschaft für Kinder- und Jugendchirurgie e.V. (DGKJCH). S1-Leitlinie Femurschaftfraktur im Kindes- und Jugendalter. Germany; 2023:006–016. https://register.awmf.org/de/leitlinien/detail/006-016;2023.

[14] Strohm PC, Schmittenbecher PP. Femoral shaft fractures in children under 3 years old: current treatment standard. Unfallchirurg. 2015;118:48–52. [cited 2026 Jan 17]. 10.1007/S00113-014-2639-7. 10.1007/s00113-014-2639-7 25480126

[15] Clavien PA, Barkun J, de Oliveira ML, Vauthey JN, Dindo D, Schulick RD, et al. The Clavien-Dindo classification of surgical complications: five-year experience. Ann Surg. 2009;250:187–196. [cited 2026 Jan 23]. 10.1097/SLA.0B013E3181B13CA2. 10.1097/SLA.0b013e3181b13ca2 19638912

[16] von Elm E, Altman DG, Egger M, Pocock SJ, Gøtzsche PC, Vandenbroucke JP. The Strengthening the Reporting of Observational Studies in Epidemiology (STROBE) statement: guidelines for reporting observational studies. Ann Intern Med [Internet]. 2007 [cited 2026 Jan 23];147:573–577. 10.7326/0003-4819-147-8-200710160-00010. 10.7326/0003-4819-147-8-200710160-00010 17938396

[17] Garraway WM, Stauffer RN, Kurland LT, O’Fallon WM. Limb fractures in a defined population. I. Frequency and distribution. Mayo Clin Proc. 1979;54:701–707. 491761

[18] Flynn JM, Skaggs DL, Waters PM. Femoral shaft fractures. In: Flynn JM, Skaggs DL, Waters PM, editors. Rockwood and Wilkins’ fractures in children. 8th ed. Philadelphia: Lippincott Williams & Wilkins/Wolters Kluwer Health; 2015.

[19] Wen Y, Wang Q, Song B, Feng W, Zhu D. External fixator versus elastic stable intramedullary nail for treatment of metaphyseal-diaphyseal junction fractures of the pediatric distal femur: a case-control study. BMC Musculoskelet Disord [Internet]. 2024;25 [cited 2026 Jan 17]. 10.1186/S12891-024-07469-Z. 10.1186/s12891-024-07469-z PMC11102165 38762453

[20] Salonen A, Laitakari E, Berg HE, Felländer-Tsai L, Mattila VM, Huttunen TT. Incidence of femoral fractures in children and adolescents in Finland and Sweden between 1998 and 2016: a binational population-based study. Scand J Surg [Internet]. 2022;111 [cited 2026 Jan 17]. 10.1177/14574969221083133. 35333132 10.1177/14574969221083133

[21] Engström Z, Wolf O, Hailer YD. Epidemiology of pediatric femur fractures in children: the Swedish Fracture Register. BMC Musculoskelet Disord. 2020;21:287. 10.1186/s12891-020-03796-z. 10.1186/s12891-020-03796-z PMC7706285 33261600

[22] Hinton RY, Lincoln A, Crockett MM, Sponseller P, Smith G. Fractures of the femoral shaft in children. Incidence, mechanisms, and sociodemographic risk factors. J Bone Joint Surg Am [Internet]. 1999 [cited 2026 Jan 17];81:500–509. 10.2106/00004623-199904000-00007. 10.2106/00004623-199904000-00007 10225795

[23] Narayanan UG, Phillips JH. Flexibility in fixation: an update on femur fractures in children. J Pediatr Orthop [Internet]. 2012 [cited 2026 Jan 17];32 Suppl 1:S10–S14. 10.1097/BPO.0B013E318255B19F. 10.1097/BPO.0b013e318255b19f 22588101

[24] Arneson TJ, Melton LJ, Lewallen DG, O’Fallon WM. Epidemiology of diaphyseal and distal femoral fractures in Rochester, Minnesota, 1965–1984. Clin Orthop Relat Res. 1988;234:188–194. 3409576

[25] Pape HC, Krettek C, Friedrich A, Pohlemann T, Simon R, Tscherne H. Long-term outcome in children with fractures of the proximal femur after high-energy trauma. J Trauma [Internet]. 1999;46:58–64 [cited 2026 Jan 17]. 10.1097/00005373-199901000-00010. 9932684 10.1097/00005373-199901000-00010

[26] Koivisto ST, Helenius I, Stenroos A, Ahola JA, Laaksonen T. Proximal femoral fractures in children: incidence, complications, and functional outcomes—a population-based study from Finland. Acta Orthop Med Journals Swed AB. 2025;96:726–34. 10.2340/17453674.2025.44752. 10.2340/17453674.2025.44752 PMC12489807 41036566

[27] Chen X, Lu M, Xu W, Wang X, Xue M, Dai J, et al. Treatment of pediatric femoral shaft fractures with elastic stable intramedullary nails versus external fixation: a meta-analysis. Orthop Traumatol Surg Res [Internet]. 2020;106:1305–1311 [cited 2026 Jan 17]. 10.1016/J.OTSR.2020.06.012. 33082120 10.1016/j.otsr.2020.06.012

[28] Benes G, Schmerler J, Harris AB, Margalit A, Lee RJ. Flexible nailing: pushing the indications for diametaphyseal lower-extremity fractures. Medicine (Baltimore) [Internet]. 2024;103:E37417 [cited 2026 Jan 17]. 10.1097/MD.0000000000037417. 10.1097/MD.0000000000037417 PMC10939545 38489726

[29] Frech-Dörfler M, Hasler CC, Haöcker FM. Immediate hip spica for unstable femoral shaft fractures in preschool children: Still an efficient and effective option. Eur J Pediatr Surg Georg Thieme Verlag. 2010;20:18–23. 10.1055/s-0029-1241177. 10.1055/s-0029-1241177 19866412

[30] Cintean R, Eickhoff A, Pankratz C, Strauss B, Gebhard F, Schütze K. ESIN in femur fractures in children under 3: is it safe? Eur J Trauma Emerg Surg [Internet]. 2022;48:3401–3407 [cited 2026 Jan 17]. 10.1007/S00068-022-01965-4. 35394142 10.1007/s00068-022-01965-4 PMC9532282

[31] Roaten JD, Kelly DM, Yellin JL, Flynn JM, Cyr M, Garg S, et al. Pediatric femoral shaft fractures: a multicenter review of the AAOS clinical practice guidelines before and after 2009. J Pediatr Orthop [Internet]. 2019 [cited 2026 Jan 17];39:394–399. 10.1097/BPO.0000000000000982. 10.1097/BPO.0000000000000982 31393292

[32] Garrett BR, Hoffman EB, Carrara H. The effect of percutaneous pin fixation in the treatment of distal femoral physeal fractures. J Bone Joint Surg - Ser B. 2011;93 B:689–94. 10.1302/0301-620X.93B5.25422. 10.1302/0301-620X.93B5.25422 21511937

[33] Schoof B, Sommerfeldt DW. Fractures around the knee in children: Epiphysiolysis and physeal fractures of the distal femur and proximal tibia. Orthopadie Springer Sci Bus Media B V. 2024;53:580–4. 10.1007/s00132-024-04528-0. 10.1007/s00132-024-04528-0 38995345

[34] Bailey MEA, Wei R, Bolton S, Richards RH. Paediatric injuries around the knee: Bony injuries. Injury Elsevier Ltd. 2020;51:611–9. 10.1016/j.injury.2019.12.033. 10.1016/j.injury.2019.12.033 32067766

[35] Persiani P, Murgia M, Ranaldi FM, Mazza O, Mariani M, Crostelli M, et al. The treatment of femoral fractures in children with cerebral palsy. Clin Ter. 2018;169:e18–22. 10.7417/T.2018.2049. 29446787 10.7417/T.2018.2049

[36] Leet AI, Shirley ED, Barker C, Launay F, Sponseller PD. Treatment of femur fractures in children with cerebral palsy. J Child Orthop [Internet]. 2009;3:253–258 [cited 2026 Jan 18]. 10.1007/S11832-009-0191-8. 19653022 10.1007/s11832-009-0191-8 PMC2726874

[37] Bukvić N, Marinović M, Bakota B, Veršić AB, Karlo R, Kvesić A, et al. Complications of ESIN osteosynthesis—Experience in 270 patients. Injury [Internet]. 2015 [cited 2026 Jan 18];46 Suppl 6:S40–S43. 10.1016/J.INJURY.2015.10.042. 10.1016/j.injury.2015.10.042 26563478

[38] Bråten M, Terjesen T, Rossvoll I. Torsional deformity after intramedullary nailing of femoral shaft fractures. Measurement of anteversion angles in 110 patients. J Bone Joint Surg Br [Internet]. 1993 [cited 2026 Jan 18];75:799–803. 10.1302/0301-620X.75B5.8376444. 10.1302/0301-620X.75B5.8376444 8376444

[39] Slongo TF. Complications and failures of the ESIN technique. Injury [Internet]. 2005 [cited 2026 Jan 17];36 Suppl 1:S78–S85. 10.1016/J.INJURY.2004.12.017. 10.1016/j.injury.2004.12.017 15652941

[40] van Cruchten S, Warmerdam EC, Kempink DRJ, de Ridder VA. Treatment of closed femoral shaft fractures in children aged 2–10 years: a systematic review and meta-analysis. Eur J Trauma Emerg Surg [Internet]. 2022;48:3409–3427 [cited 2026 Jan 10]. 10.1007/S00068-021-01752-7. 34338819 10.1007/s00068-021-01752-7 PMC9532337

